# Underlying mechanisms of evasion from NK cells as rationale for improvement of NK cell-based immunotherapies

**DOI:** 10.3389/fimmu.2022.910595

**Published:** 2022-08-12

**Authors:** Barbara Seliger, Ulrike Koehl

**Affiliations:** ^1^ Institute of Medical Immunology, Martin Luther University Halle-Wittenberg, Halle (Saale), Germany; ^2^ Fraunhofer Institute for Cell Therapy and Immunology, Leipzig, Germany; ^3^ Institute of Clinical Immunology, University of Leipzig, Leipzig, Germany; ^4^ Institute of Cellular Therapeutics, Hannover Medical School, Hannover, Germany

**Keywords:** NK cells, immune escape, immunotherapy, tumor, HLA

## Abstract

Natural killer (NK) cells belong to the family of innate immune cells with the capacity to recognize and kill tumor cells. Different phenotypes and functional properties of NK cells have been described in tumor patients, which could be shaped by the tumor microenvironment. The discovery of HLA class I-specific inhibitory receptors controlling NK cell activity paved the way to the fundamental concept of modulating immune responses that are regulated by an array of inhibitory receptors, and emphasized the importance to explore the potential of NK cells in cancer therapy. Although a whole range of NK cell-based approaches are currently being developed, there are still major challenges that need to be overcome for improved efficacy of these therapies. These include escape of tumor cells from NK cell recognition due to their expression of inhibitory molecules, immune suppressive signals of NK cells, reduced NK cell infiltration of tumors, an immune suppressive micromilieu and limited *in vivo* persistence of NK cells. Therefore, this review provides an overview about the NK cell biology, alterations of NK cell activities, changes in tumor cells and the tumor microenvironment contributing to immune escape or immune surveillance by NK cells and their underlying molecular mechanisms as well as the current status and novel aspects of NK cell-based therapeutic strategies including their genetic engineering and their combination with conventional treatment options to overcome tumor-mediated evasion strategies and improve therapy efficacy.

## General features of NK cells

Natural killer (NK) cells are cytotoxic innate immune cells that were first described in 1973 by E. Klein and colleagues (1). They originate from multipotent hematopoietic stem cells (HSC) in the bone marrow (BM) and undergo different developmental stages gradually acquiring the expression of distinct surface markers defining the commitment to the lymphoid/NK cell lineage. Maturation of human NK cells is characterized by a loss of CD34 and c-KITC (CD117) expression followed by a sequential upregulation of CD94, CD16 and killer cell immunoglobulin-like receptors (KIRs) (2). NK cells comprise 5-10% of peripheral blood mononuclear cells (PBMCs), but they are also found with a variable frequency in various lymphoid and non-lymphoid tissues including BM, liver, lung, skin, kidney and spleen (3). NK cells have the capacity to form cytoplasmic lytic granules containing perforin and granzymes and produce a large number of cytokines, in particular interferon (IFN)-γ, but also proinflammatory and immune suppressive cytokines, such as tumor necrosis factor (TNF)-α, interleukin (IL)-10, chemokines and various growth factors like granulocyte-macrophage stimulatory factor (GM-CSF), granulocyte-stimulating factor (G-CSF) and IL-3. They exert their cytotoxic activity by distinct mechanisms, including the release of granzymes and perforin, secretion of IFN-γ and TNF-α, the expression of the FasL/Fas or TNF-related apoptosis-inducing ligand (TRAIL)/TRAIL receptors and the antibody-dependent cell-mediated cytotoxicity (ADCC) *via* Fc receptors (CD16) recognizing antibodies bound to antigen-coated (tumor) cells (4–6). Based on their cytolytic function, NK cells play a key role in the first line of immune defense and are able to directly eliminate tumor or pathogen-infected cells. In this context it is noteworthy that NK cells have safety features, rarely elicit autoimmunity and promote immune homeostasis.

NK cells arise and progressively evolve from a limited diversity to highly differentiated and heterogeneous phenotypes, which are dictated by genetic factors and environmental stimuli, such as pathogen exposure, leading to distinct functions (7). In PBMCs, NK cells are generally subdivided into two major subsets based on their differential expression of CD56: (i) CD56^bright^, CD94^+^, CD16^-^ NK cells, which are less abundant in PBMCs, are poorly cytotoxic, but produce high amounts of IL-1β, IFN-γ, IL-2, IL-12, IL-15, IL-18 and TNF-α upon stimulation, extensively proliferate in response to DC-derived cytokines and can extravasate from the circulation into tissues and (ii) CD56^dim^, CD16^+^ and KIR^+^ NK cells, which have a low proliferative capacity, but high cytotoxic activity accounting for most of the circulating NK cells (8, 9). Furthermore, terminally differentiated CD57^+^ and adaptive NKG2C^+^CD57^+^ NK cells exist (10). Also, the discovery of memory-like NK cells being able to mount a robust secondary immune response upon activation has expanded the understanding of this innate immune cell population over the past decade (11, 12).

With the possibility of the in depth characterization of immune cell subpopulations by high-dimensional transcriptional and phenotypic profiling using (single cell) RNA-sequencing (RNA-seq) and mass cytometry an unexpected NK cell diversity was identified across different organs within individual donors regarding their function, maturation and interaction with stromal cells, which also provide a new framework for the analyses of NK cell responses under physiologic and pathophysiologic conditions (13–17). Interestingly, the diversity of NK cells was found both in the immune cell infiltrate of tissues and in peripheral blood (17, 18).

## NK cell receptors and NK cell activity

NK cells are tightly regulated by a dynamic balance of transduced signals mediated by the physical interaction with adjacent cells. They express a number of germline-encoded activating and inhibitory receptors as well as cytokine and chemokine receptors on the cell surface, which influence the NK cell function, but knowledge of how these receptors convey signals and affect NK cell biology is still limited. There is evidence of a balance between activating and inhibitory receptors, which control the activity, cell diversity and function of NK cells (19, 20). These constitutively expressed NK cell receptors comprise non-HLA-specific receptors, HLA-specific receptors and homing receptors (20), and recognize their corresponding ligands expressed on the cell surface of target cells such as tumor cells or virus-infected cells (21), as summarized in **Table 1**.

**Table 1:** Major NK cell receptors and their ligands.

A: Activating NK cell receptors and their ligands		
NK cell receptor type	Name	Ligands
non-HLA	NKp30 (CD337)	B7-H6, BHG6/BAT3, galectin
	NKp44 (CD336)	MLL5-Nidogen-1, PDGF-DD, PCNA
	NKp46 (CD335)	viral HA and HN, properdin
	NKG2D (CD314)	MICA, MICB, ULBPs
	FcyRIII (CD16)	IgG
	TLR3/9	microbial constituents, CpGs
	CD2	CD58
	α-integrin	vascular endothelial growth factor
	DNAM1 (CD226) 2B4	nectin2 (CD112), PVR (CD155) CD48
HLA I	KIR3DS2	HLA-C C1, HLA-A* 11:01
	CD94/NKG2C	HLA-E
	KIR2DS4	HLA-F, HLA-C, HLA-A* 11
	KIR2DS5	HLA-C C2?
	KIR3DS1	HLA-B* 51, HLA-F
	2B4	CD48
	KIR3DS1 (CD158b)	HLA-C2
	KIR2DL4 (CD158d)	HLA-G
B: Inhibitory NK cell receptors and their ligands		
NK cell receptor type	Name	Ligands
HLA I	NKG2A (CD159a/CD94)	HLA-E
	KIR2DL1, DL2, DL3 (CD158a,b)	HLA-C, HLA-B
	KIR3DL1, DL2 (CD158e,k)	HLA-A, -B or -F
	ILT2/LIR-1 (CD85J)	HLA-G, different HLA class I allotypes
	LAG-3	MHC class II
non-HLA	TIM-3	galectin-9, HMGB1, CEACAM1
	PD-1 (CD279)	PD-L1, -L2, CD273
	TIGIT	PVR (CD155, CD274), nectin2 (CD112), nectin4, CD113
	Siglec 7 (CD328)	ganglioside DSGb5
	LAIR-1	collagen

The major NK cell receptors and their ligands are summarized as recently reviewed (21–23).

Next to CD16 (FcγRIIIA), which interacts with Fc fragments of several IgG subclasses, triggering the ADCC (24), the natural cytotoxicity receptors (NCR) NKp30 (CD337), NKp44 (CD336), NKp46 (CD335), NKp80, DNAM-1 (CD226) and NKG2D (CD314) are the major activating receptors and are able to recognize induced self-ligands that are downregulated on healthy cells and highly expressed on tumor cells (25). There are a number of HLA class I-specific activating NK cell receptors (NKR) that recognize the non-classical HLA class I antigens HLA-E and HLA-F or epitopes shared by distinct HLA class I allotypes. For the activating receptor KIR2DS3, the ligand is still unknown. Other NK cell activating receptors include SLAMs, CD18, CD2 and the toll-like receptor (TLR) 3/9 (26, 27).

The primary inhibitory receptors on the cell surface of NK cells represent members of the killer cell immunoglobulin-like receptor (KIR) family, which consists of 14 polymorphic receptors. The different inhibitory KIRs can recognize classical HLA class I antigens, but for KIR2DL5 no ligand has yet been identified. Other inhibitory receptors include NKG2A, a member of the C-type lectin family, which heterodimerizes with CD94 and binds to the HLA-E antigen, the immunoglobulin-like receptor superfamily B member 1 (LILRB1, ILT2, CD85j), the T cell immunoglobulin and mucin domain containing molecule 3 (TIM-3), T cell immunoreceptor with Ig and immunoreceptor tyrosine-based inhibition motif (ITIM) domains (TIGIT) (28–30), CD161, SIGLEC7, SIGLEC9, programed death receptor 1 (PD-1) and lymphocyte-activation gene (LAG-3) (31). These inhibitory receptors regulate the activation status and anti-tumoral immunity of NK cells by suppressing effector functions and augmenting Treg activity (32).

MHC class I molecules are ligands of the inhibitory receptors of NK cells thereby providing signals to self-tolerance resulting in NK cell inactivation and the discrimination between healthy, “self” and “non-self” cells including tumor or virus-infected cells. However, tumor or pathogen-infected cells often lack or downregulate MHC class I surface antigens, which results in an escape from recognition by CD8^+^ cytotoxic T lymphocytes (CTL). In contrast, these MHC class I-negative cells could be recognized and eliminated by NK cells *via* the missing self-mechanism (“missing-self recognition”). However, the NK cell activation requires additional signals to induce self changes, e.g. by virus-encoded ligands or ligands upregulated by cellular stress, by DNA damage and alterations of suppressor genes (33) leading to the so-called “induced self-recognition” (34). Activated NK cells can eliminate target cells either directly *via* NK cell-mediated cytotoxicity or indirectly *via* proinflammatory cytokine-mediated killing by TNF-α and IFN-γ. In addition to the interaction with tumor and pathogen-infected cells, NK cells could crosstalk with other immune cells, like macrophages, T lymphocytes and different dendritic cell (DC) subpopulations (35, 36). Over the last decade, the functional links between NK cells and myeloid cells have been broadly analyzed. This cooperative interaction triggers the innate and adaptive immune responses by stimulating the survival, maturation and tumor infiltration of DCs leading to “DC editing” (37–39). Vice versa, macrophages could shape NK cell differentiation and function (40).

## NK cells as critical players for tumor immune surveillance

The primary role of NK cells is the recognition and elimination of tumor cells or virus-/pathogen-infected cells as the first line of defense against initiation of tumor formation and pathogen invasion without prior sensitization (41). Evidence for this hypothesis is an increased tumor incidence in human and experimental models with impaired NK cell function (42). NK cells are educated and licensed by inhibitory receptors that recognize classical MHC class I molecules, but could recognize MHC class I-deficient cells, which are then eliminated (43). Thus, NK cells are activated by tumor cells due to the decreased expression of MHC class I on tumor cells through the lack of inhibitory signals and by the induction of activating NK cell receptor ligands through their “missing-self” program (44) leading to productive cytotoxic responses. An additional major pathway involved in NK cell-mediated cytotoxicity is the FasL/Fas interaction, which provides a death signal to target cells leading to apoptosis. The activating receptor NKG2D on NK cells recognize the MHC class I-related surface proteins MICA and MICB as well as the UL-16-binding proteins (ULBPs; ULBP1-6), which are often upregulated in e.g. tumor cells countermanding any inhibitory signals and inducing NK cell-mediated cytotoxicity (45, 46).

## Composition of the tumor microenvironment and NK cells

Detailed analysis of the tumor microenvironment (TME) in different cancer types demonstrated a complex network of immune effector cells, such as CTL and NK cells, but also immune suppressive cells, like regulatory T cells (Tregs), tumor-associated macrophages (TAMs), regulatory γδ T cells, myeloid-derived suppressor cells (MDSCs), soluble factors, extracellular matrix (ECM) components as well as suppressive molecules expressed on tumor cells. The interaction between the different immune cell subpopulations in the TME with tumor cells is diverse and orchestrated by the presence of specific chemokines and cytokines recruiting different immune suppressive cells into the TME and modulating immune effector cells, which is associated with tumor progression (47). This complex interplay is also shaped by changes in the metabolic activity of immune, stromal and tumor cells (48).

The distribution of NK cells is highly dynamic. Circulating NK cells can migrate into tissues *via* the expression of a broad number of receptors that control this recruitment (31). In different tissues, NK cells display specific phenotypic and functional features, which are altered by the physiologic and pathophysiologic micro-milieu. To reach the solid tumors, NK cells extravasate from the blood and traverse the ECM and the tumor stroma. In the tumor bed, NK cells are able to control tumor growth and metastasis (49). However, NK cell responsiveness is often reduced by the immune suppressive TME.

Although NK cells have been demonstrated to infiltrate into primary solid tumors, metastases and even into tumor-draining lymph nodes, the frequencies of NK cells in solid tumors were lower when compared to adjacent tissues and less abundant regarding the numbers of CD4^+^ and CD8^+^ T cells and B lymphocytes. The degree of NK cell infiltration in tumors is influenced by several factors (50), such as tumor localization, nature of cancer cells and expression of chemokine receptors/chemokines (51). In addition, NK cells recruited to the tumor core had a reduced cytotoxic potential compared to NK cells from normal tissues and are often associated with an unfavorable condition for survival (52, 53). The clinical relevance of tumor-infiltrating NK cells, e.g. their correlation with the patients’ survival, depends on the expression of ligands for their receptors and is accompanied by a high variability of the different NK cell populations in distinct tumor entities (54). For example, NK cell frequencies are associated with an altered patients’ survival in many tumor entities. NK cells highly infiltrating renal cell carcinoma (RCC) were dysfunctional in *ex vivo* cultures (14, 55, 56) (**Table 2**) and showed an increased expression of inhibitory receptors and a downregulation of activating receptors. Furthermore, low numbers of NK cells in head and neck squamous cell carcinoma (HNSCC) were associated with insufficient tumor elimination, while higher numbers of NK cells at the tumor site correlated with an increased patients’ survival. Comparable results were also shown for colorectal carcinoma (CRC), gastric and esophageal cancer. Concerning non-small cell lung cancer (NSCLC), NK cells are less frequent in tumor tissues compared to normal lung epithelium, overexpress NK cell inhibitory receptors and show a CD56^bright^ perforin^low^ phenotype. The number of NK cells in NSCLC is of clinical relevance and linked to the tumor size, smoking history and a bad patients’ prognosis (31, 68).

**Table 2:** NK cell infiltration and its prognostic value.

Tumor	Method	NK cell identification	Tumor tissue	Infiltration tumor vs. metastasis/normal cells	Prognostic factor/survival	Reference
breast cancer	FC	CD3^-^ CD56^+^	primary	down	OS	(57, 58)
colorectal carcinoma	IHC/FC	NKp46^+^	primary/metastasis	down	OS, DFS	(59)
gastric and colorectal cancers	FC	CD3^-^ CD56^+^	metastasis	down	OS	(60)
endometrial cancer	FC/IHC	CD3^-^ CD56^+^	primary	down	DFS	(54, 61)
esophageal cancer	FC	CD3^-^ CD56^+^	primary	down		(62)
gastric cancer	FC	CD3^-^ CD56^dim^ CD57^+^	primary	down	OS	(62)
melanoma	FC	CD3^-^ CD56^dim^	lymph node	up		(63)
non-small-cell lung carcinoma	FC	CD3^-^ CD56^+^	primary	down	tumor size, OS	(50, 64, 65)
renal cell carcinoma	FC/IHC	CD3^-^ CD56^+^ NKp46	primary	up	metastasis different, OS	(66)
several	FC	CD3^-^ CD56^+^	primary	diverse		(50)
lung adenocarcinoma	IHC	CD57^+^	primary	down	OS	(67)

DFS, disease free survival; FC, flow cytometry; IHC, immunohistochemistry; OS, overall survival.

Bioinformatics of large RNA-seq datasets from The Cancer Genome Atlas (TCGA) revealed not only a link between NK cell numbers and patients’ survival (31), but also identified a NK cell signature of 13 genes, which makes it possible to determine NK cell abundance across different tumor types and offers novel opportunities for NK cell-based treatment in specific cancer conditions (69, 70). Thus, strategies that increase the recruitment and activation of NK cells in tumors would be a suitable approach to enhance anti-tumor efficacy (71).

## Impaired NK cell functions due to intrinsic mechanisms

Studies of different tumor entities demonstrated that the function of intra-tumoral NK cells is impaired, which might be due to aging, genetic defects and chronic infections (72–74), but also due to continuous exposure to tumor antigens (75–77). Tumor escape from NK cell-mediated immune surveillance could be due to impaired anti-tumor NK effector mechanisms, such as reduced production of proinflammatory cytokines, e.g. IFN-γ and TNF-α, proliferation and cytotoxicity due to a diminished expression of effector molecules, like perforin and granzymes. Various solid and hematopoietic cancers demonstrated a downregulation of the activating receptors NKp30, NKG2D, NKp46 and CD16 and an increase of soluble NKG2D ligands sMICA/B shed from the tumor cell surface, but high expression levels of the inhibitory receptor CD94/NKG2A, resulting in impaired NK cell cytotoxicity (78). This was associated with a poor prognosis of patients with breast cancer, chronic lymphocytic leukemia (CLL), ovarian cancer and acute myeloid leukemia (AML) (79). The presence of NK cells in the TME and higher expression levels of CD56, CD57, NKp30 or NKp46 at the tumor site were associated with a favorable patients’ prognosis, while low NK cell numbers correlated with an increased risk of cancer recurrence after resection, and a reduced patients’ survival (80). In NSCLC, overexpression of inhibitory NK cell receptors and a reduced number of NK cells was associated with a poor patients’ outcome (64, 81) which was accompanied by a reduced cytotoxicity and promotion of tumor evasion. Next to the distinct expression pattern of NK cell receptors, the programmed death receptor PD1 has been well characterized as an exhaustion marker for T cells, but also for NK cells (82). The same applies to TIGIT, which is also associated with NK cell exhaustion (83). It is noteworthy that actin cytoskeleton remodeling and fragmented mitochondria in the cytoplasm of tumor-infiltrating NK cells can also lead to immune suppression (84, 85).

## Impaired NK cell function due to extrinsic mechanisms

It is generally accepted that the TME shapes the innate as well as the adaptive immune responses, which are variable between distinct tumor types due to differences in the composition of infiltrating immune cells and soluble constituents. It is noteworthy that the critical function of NK cells to induce an effective anti-tumor immunity is a successful interaction between NK cells and DCs, and the production of chemokines. Both processes are negatively influenced by unique locoregional characteristics, in particular cellular and soluble components of the TME, which are associated with an immune escape due to a lack effector responses thereby promoting tumor cell metastasis (75–77). The chemokine milieu in the TME consists of reduced expression of CXCL2, CX_3_CL1, CXCL1 and CXCL8 thereby attracting CD56^dim^ NK cells and an increased CXCL9/10, CCL5 and CXCL19/21 expression driving the homing of CD56^bright^ NK cells toward the stromal compartment (86).

Solid tumors often showed a high oxygen consumption, a low pH in the TME due to higher concentrations of lactate and a disorganized vascularization leading to hypoxia as well as an altered expression of genes involved in the regulation of metabolic processes. An acidic microenvironment (87) and a permanent or transient hypoxia leading to an upregulation of the transcription factor HIF-1α (88) due to the restricted access to nutrients and oxygen mediated by changes in the vascularization have been demonstrated to downregulate the expression of activating NCRs, reduce NK cell cytotoxicity and survival, which downregulates NK cell anti-tumor responses (89). This could be reverted by e.g. the treatment with an inhibitor of HIF-1α (89). In addition, NK cells may not penetrate into solid tumors including in low MHC class I-expressing tumors or once within the tumors become anergic or exhausted. Increased H_2_O_2_ levels lead to a decrease in the infiltration of CD56^dim^ NK cells and impaired ADCC. Furthermore, NK cells in tumors can also acquire proangiogenic functions by secretion of vascular endothelial growth factor (VEGF), angiogenin and matrix metalloproteinases (MMPs) (90, 91). Although a proangiogenic NK cell phenotype has been identified, the potential of proangiogenic NK cell-driving tumor progression has not yet been analyzed in detail. However, tumor endothelium might improve NK cell recruitment to the tumor site as an indirect mechanism of targeting myeloid cells affecting NK cell recruitment and function (92).

Several immune suppressive cells, like MDSC, TAMs and Tregs negatively interfere with NK cell activation. This has been attributed to immune modulatory molecules present in the TME, such as indolamine 2, 3-deoxygenase (IDO) activity and transforming growth factor (TGF)-β, which can be secreted by MDSC, Tregs and anti-inflammatory macrophages. Additionally, IL-1β secreted by 6-sulfo LacNAc DCs induces cell apoptosis (93), while Tregs could also suppress NK cells by deprivation of IL-2 (94). Several other factors produced by tumor or tumor-associated cells, like prostaglandin E2, extracellular adenosine, IL-10 and IL-6, further directly or indirectly prevent NK cell activation (95). During infection and tumorigenesis, macrophages can modulate NK cell function by direct cell-to-cell contact or due to secretion of the cytokines IL-18, IL-12 and TGF-β (96). TGF-β modulates NK cell function *via* a decrease of NKG2D levels and CD16-mediated ADCC in tumors by impairing the cytotoxic potential as demonstrated in *in vivo* and *in vitro* co-culture experiments. In addition, TGF-β affects the expression of chemokine receptors thereby preventing NK cell recruitment as well as the NK cell metabolism by inducing a reduced glycolysis and oxidative phosphorylation that inhibits NK cell effector function. NK cell dysfunction has been associated with the inactivation of the glycogen synthase kinase-3 (GSK3). In contrast, IL-15 is chemotactic for NK cells and maintains NK cell activation by suppressing tumor escape mechanisms (97). However, sustained persistence of IL-15 in the TME could induce the expression of the cytokine-inducible SH2-containing protein, an IL-15 inducible IL-15 signaling inhibitor, leading to the degradation of IL-15R. This is associated with a diminished responsiveness of NK cells to IL-15 (98).

## Strategies of tumor cells evading NK cell recognition

As described above, NK cells preferably recognize and kill malignant cells. But there exist many different strategies of tumors to directly evade NK cell recognition. On the one hand, these include the prevention of NK cell recruitment into tumors by physical barriers (laminin and collagen) of tumors or by preferential recruitment of immature NK cells *via* a chemokine gradient. On the other hand, tumors dampen the NK cell activation and effector function by a decreased expression of ligands for the activating NKRs or by generation of soluble activating receptor ligands, which block recognition. In contrast, inhibitory molecules, like the non-classical HLA class I molecules HLA-G and -E, Nectin-4 or PVR and inhibitory immune checkpoint (ICP) ligands, are often overexpressed in tumors thereby impairing not only T cell, but also NK cell responses (20, 21). High levels of HLA-E were found in many solid tumors and its overexpression correlated with a poor prognosis and NK cell exhaustion (99, 100), while the innate immunity is regulated by the engagement of HLA-G with the NK cell receptor KIR2DL4 or ILT2 (101, 102) leading to a reduced cytotoxicity. Many tumors express the MHC class I chain-regulated polypeptide A (MICA) and MICB, known as ligands for the activating receptor NKG2D on NK cells. However, tumors frequently shed MICA and -B thereby removing an activation signal and creating a soluble ligand, which can block the NK cell cognate receptors (103, 104). Thus, classical, non-classical as well as HLA class I-related molecules play a key role in NK cell functionality by either leading to immune escape or immune recognition. Characterization of these immune escape mechanisms represent the rational for the development of NK cell-based immunotherapies.

## Different strategies to revert immune surveillance by NK cells-antibody-based approaches

Since NK cell anti-tumor function is frequently impaired in tumor patients, restoring their function is an obvious therapeutic option. Indeed, there exist different approaches to restore the anti-tumor surveillance of NK cells (105). Agents that enhance NK cell function, like immune modulatory drugs, various stimulatory cytokines, STING agonists and TGF-β inhibitors have been recently summarized (106). In addition, a number of mAbs directed against key ICP ligands and their receptors have been designed, which prevent NK cell inactivation by e.g. decreasing inhibitory factors or increasing factors, which boost NK cell function. Recently, a humanized anti-NKGA mAb (monalizumab) has been developed, which exerts *in vitro* and *in vivo* anti-tumor efficacy as a single agent or with other therapeutics (107). The inhibition of NKG2A restores the cytotoxic activity against HLA-E-expressing target cells as well as the NK cell-dependent maturation of monocyte-derived DC and reduces the secretion of immune suppressive cytokines.

Major ICP-targeted therapies that affect NK cell-mediated anti-tumor immune responses are the immune checkpoint inhibitors (ICPis) PD1/PD-L1 and CTLA4. PD1 has been shown to be mainly expressed on T, B and myeloid cells, but also on about 25% of NK cells in healthy donors, but the molecular mechanisms leading to PD1 expression have not yet been identified (108). PD1 can also be expressed on tumor infiltrating NK cells of patients with different solid tumors (109). Blockade of PD1/PD-L1 interaction can enhance NK cell activity both *in vitro* as well as in animal models due to an enhanced ADCC-induced anti-tumor function leading to an increased tumor control. Moreover, NK cells play also a role in response to treatment with agonistic anti-CD137/4-1BB antibodies (Abs) (110). CD137 is expressed on primed NK cells, which upon ligation provides a powerful costimulatory signal (111). The addition of agonistic Abs increased NK cell proliferation and a synergistic effect was found between IL-15 and IL-21 upon CD137 engagement and the presence of APCs. Thus, CD137 triggering contributes to NK cell activation (112). These data suggest that restoring of the NK cell function by co-targeting immune modulatory pathways might be an important therapeutic strategy to prevent tumor immune escape.

Since intra-tumoral activated NK cells are often characterized by overexpression of TIGIT, which competes with the activating NK cell receptor DNAM1, TIGIT blockade might also be a promising approach (113) and has been described to increase patients’ response (114). However, TIGIT and the activating receptor DNAM1 have CD155 as ligand suggesting a complex of CD155-mediated immune regulation *via* these receptors. Human tumor cells could express both membranous and soluble CD155. The latter binds preferentially and with a higher affinity to DNAM1 thereby inhibiting the DNAM1-mediated anti-tumor activity of NK cells (115). Recent studies also focused on increasing the infiltration and recruitment of NK cells by inhibiting soluble factors secreted by tumor cells, e.g. TGF-β (116). Furthermore, antibodies targeting the proteolytic site of MICA shedding can promote NK cell-driven tumor immunity (117).

In addition, Abs directed against inhibitory KIRs are potential therapeutic candidates, which might have fewer side effects compared to other therapeutic approaches. Recently, a humanized anti-NKG2A mAb monalizumab has been developed, which is explored in clinical trials (NCT02643550, NCT02921685). Other trials are addressing IPH4102 as an anti-KIR3DL2 (NCT02593045), lirilumab as an anti-KIR2DL1-3 (NCT01687387) antibody as well as different Abs directed against the PD1/PD-L1 axis.

## Benefit and limitation in clinical NK cell-based immunotherapies – Adoptive cell transfer-based approaches

The translation of *in vitro* and *in situ* results of modulating NK cell activity and function into clinical concepts has been challenging and was investigated in a number of clinical trials. Over the past decades, considerable progress has been made in NK cell-based immunotherapies in haploidentical stem cell transplantation (haploSCT) or in the non-transplant setting, since allogeneic NK cells contribute to the graft versus leukemia/tumor effect (GvL/GvT) with generally no or only marginal graft versus host disease (GvHD) compared to allogeneic T cells (118–120). There are several sources for NK cells. They can be obtained from (i) healthy donors *via* leukapheresis followed by immunomagnetic purification (CD3-depleted, CD56-enriched), (ii) cord blood or (iii) induced pluripotent stem cell (iPSC) and administered unstimulated or cytokine-activated and expanded, respectively. After the first clinical trials in 2004 and 2005 using IL-2 activated donor NK cells, performed in parallel in Europe and the USA (121, 122), multiple clinical trials over the last 1.5 decades showed safety and feasibility of adoptive NK cell transfer for various hematological and oncological diseases, respectively (123). Despite the overall clinical benefit regarding GvL/GvT effect without GvHD, adoptive NK cell therapies are hampered by tumor immune escape mechanism, such as blocking of NKG2D by soluble MICA (124), exhaustion of NK cells in the immune-suppressive tumor microenvironment (125) and limited persistence of NK cells. In addition to the historical use of IL-2 for both *ex vivo* expansion of the NK cells during manufacturing and *in vivo* therapy, stimulation of NK cells with IL-12, IL-15, IL-18 and IL-21 enhanced cytotoxicity, successfully generated donor memory-like NK cells with enhanced persistence and improved anti-leukemia response. This could be demonstrated impressively in 4/8 pediatric patients with AML in a current clinical trial (126). Cytokine combinations are increasingly used for optimized manufacturing protocols (127). Nevertheless, the optimal cytokine cocktail after adoptive NK cell transfer to improve cell expansion remains still unclear. Very recently, it has been shown in clinical trials that systemic IL-15 resulted in reduced clinical activity (128). The authors hypothesized that IL-15 promotes recipients CD8^+^ T cell activation that finally leads to donor NK cell rejection.

Other trials are using NK cell subpopulations. Especially, cytomegalovirus (CMV) infection is one powerful stimulus promoting the functionality and phenotype of NK cells expressing the HLA-specific activating receptor CD94/NKG2C (129). Therefore, clinical protocols are currently developed based on the mechanisms underlying the generation of adoptive NK cells that involve NKG2C triggering to efficiently expand NKG2C^+^ NK cells for therapy. Interestingly, adoptive NK cells appear to be resistant to MDSC and Treg suppression thereby providing them with a further advantage compared to CAR T cells for their use as therapeutics. Another benefit is the availability of NK cells for therapy from distinct sources. Multiple other approaches are ongoing to restore NK cell activity and reach long-lasting effects. These include the blockade of inhibitory receptors, blocking soluble activating receptors, combinational therapies with immune checkpoint inhibitors (130) and genetic engineering of the NK cells (131). In addition, cytokine-activated NK cells with upregulated NCRs and NKG2D are partly able to overcome tumor immune escape by restoring NKG2D-mediated NK cell cytotoxicity *via* scavenging of plasma MICA as demonstrated for neuroblastoma and head and neck cancer, respectively (124, 132).

## Engineered NK cell-based immunotherapies

Genetic modification of immune effector cells has been demonstrated to be a promising strategy for the treatment of advanced cancers refractory to conventional therapies. In particular, chimeric antigen receptor (CAR) targeting cell surface antigens provide a suitable tool to increase the efficacy of effector cells. CARs are genetically engineered proteins composed of an extracellular domain specific for the respective/selected target antigen, a transmembrane domain and an intracellular signaling domain responsible for the transduction of the activating signal. During the last two decades, the CAR technology has been developed as next generation immunotherapeutic approach reaching impressive clinical results in two hematological disorders, the acute lymphoblastic leukemia and diffuse large B cell lymphoma. This led to more than 800 clinical trials worldwide (clinicaltrials.gov) (133) as well as to five approved CAR T cell products, the first four targeting CD19 and the last one directed against BCMA, respectively (134). In a similar way, engineered CAR NK cells redirected against several cancer epitopes including hematological and tumor targets resulted in improved NK cell cytotoxicity (135–138). Moreover, in addition to use the intracellular CAR T cell signaling, DAP10 and DAP12 give rise for more improvement for CAR NK cell cytotoxicity (139). There is a clear advantage of CAR NK cells over CAR T cells, since NK cells can be obtained from allogeneic donors, do not induce a cytokine storm, persist for more than one year and can be applied to the patients without development of GvHD and thus represent an “off the shelf” product for the treatment of patients (105, 137, 140). Another challenge is to overcome the high manufacturing costs of personalized autologous CAR T cell products by using one allogeneic CAR NK cell product for multiple applications in various patients. To date, more than 35 clinical trials using CAR NK cells are conducted (clinicaltrials.gov) against several cancer epitopes, such as CD19, CD19/22, CD33, CD7, HER2, MUC1, PDL1, NKG2D ligand, BCMA, ROBO1, PSMA, mesothelin and others using CAR NK cells from different sources, primary human NK cells, cord-blood derived and iPSC-derived NK cells as well as CARs from the cell line NK92 (141, 142).

While most of the trials are performed in China and USA, currently a phase I trial, CAR2BRAIN using lentiviral transduced CAR NK92 cells redirected against the human epidermal growth factor 2 (HER2, ErbB2) for treatment of recurrent patients with glioblastoma is conducted in Europe, in Frankfurt, Germany. The very well recognized study of Katy Rezvani, USA, employed cord-blood derived CAR NK cells redirected against CD19 for B cell malignancies. The promising results showed clinical responses in 8 out of 11 patients with no sign of cytokine release syndrome or neurotoxicity (143). Next to conventional CARs, additional genetic modifications are currently explored to enhance NK cell activity and homing into the tumor. Preclinical studies demonstrated an improvement of tumor cell infiltration through transgene expression of chemokine or adhesion receptors (144). Furthermore, the integration of the autocrine growth factor IL-15 as a down-stream cassette has been used, which led to an improved life span and persistence of those CAR NK cells in all patients (145). The overall cytokine and chemokine profile clearly differ between CAR T and CAR NK cells and supports the observation that allogeneic CAR NK cells do not contribute to any severe side effects, like cytokine release syndrome and toxicity. Nevertheless, NK cells are considered hard-to-engineer and hard-to expand compared to T cells. Recently, a novel viral envelope derived from the baboon endogenous virus (BaEV) showed superior efficacy as compared to other lentiviral envelope proteins to successfully genetically manipulate human NK cells (146, 147).

Finally, the question arises how to further improve both anti-tumoral activity, cytotoxicity and homing of CAR NK cells in the TME, which led to combinational therapies with ICPis. In the end of 2021, two clinical trials were started: (i) a phase II study using irradiated PD-L1 CAR-NK cells plus pembrolizumab for recurrent/metastatic gastric or head and neck cancer (NCT04847466) and (ii) FT576 (iPSC derived CAR NK cells) as monotherapy and in combination with daratumumab in subjects with relapsed/refractory multiple myeloma (NCT05182073), respectively.

## Conclusions

The enhancement of NK cell activity represents an important approach to control cancer growth. The increased understanding of the NK cell biology has led to the development of NK cell-based strategies to control tumors. New ways to enhance the NK cell targeting, their activation and cytolytic function are required, since the NK cells are becoming dysfunctional in the immune suppressive TME. Despite the potential of NK cell-based therapies it has become obvious that for the design of effective strategies using NK cells in the clinics, a detailed knowledge of NK cell receptors, NK cell subpopulations, tissue-specific NK cells and memory-like NK cells is required. Furthermore, the NK cell heterogeneity might influence the efficacy of NK cell-based therapies. Some preclinical and clinical studies suggest multifaceted opportunities of the implementation of NK cells for the treatment of cancer patients using combination therapies, which will lead to further clinical advances.

## Author contributions

All authors listed have made a substantial, direct, and intellectual contribution to the work and approved it for publication.

## Conflict of interest

The authors declare that the research was conducted in the absence of any commercial or financial relationships that could be construed as a potential conflict of interest.

## Publisher’s note

All claims expressed in this article are solely those of the authors and do not necessarily represent those of their affiliated organizations, or those of the publisher, the editors and the reviewers. Any product that may be evaluated in this article, or claim that may be made by its manufacturer, is not guaranteed or endorsed by the publisher.

## Abbreviations

Ab, antibody; ADCC, antibody-dependent cell-mediated cytotoxicity; ALL, acute lymphoblastic leukemia; BM, bone marrow; CAR; chimeric antigen receptor; CLL, chronic lymphocytic leukemia; CMV, cytomegalovirus; CR, complete revision; DC, dendritic cell; DFS, disease free survival; ECM, extracellular matrix; EGF-R, epidermal growth factor receptor; EV, extracellular vesicle; FC, flow cytometry; FDA, Food and Drug Administration; G-CSF, granulocyte-stimulating factor; GM-CSF, granulocyte-macrophage stimulating factor; GMP, good medical practice; Had, graft-versus-host disease; HLA, human leukocyte antigen; HSC, hematopoietic stem cells; HSCT, hematopoietic stem cell transplantation; ICPi, immune checkpoint inhibitor; IDO, indolamine 2, 3-deoxygenase; IFN, interferon; IL, interleukin; iPSC, induced pluripotent stem cell; KIR, killer cell immunoglobulin-like receptors; LAG, lymphocyte-activation gene; mAb, monoclonal antibody; MDSC, myeloid-derived suppressor cell; MHC, major histocompatibility complex; MIC, MHC class I-related; MMP, matrix metalloproteinase; NCR, natural killer receptor; NK, natural killer; OS, overall survival; PBMNC, peripheral blood mononuclear cell; PD1, programmed death; PD-L1, programmed death ligand 1; RNA-seq, RNA-sequencing; TAM, tumor-associated macrophages; TCR, T cell receptor; TGF-β, transforming growth factor β; TIGIT, T cell immunoglobulin and immunoreceptor tyrosine-based inhibitory motif; TMB, tumor mutational burden; TME, tumor microenvironment; TNF; tumor necrosis factor; TRAIL, TNF-related apoptosis inducing ligand; Treg, regulatory T cell; ULBP, UL-16 binding protein; VEGF, vascular endothelial growth factor

## Glossary

**Table:**

Ab	antibody
ADCC	antibody-dependent cell-mediated cytotoxicity
ALL	acute lymphoblastic leukemia
BM	bone marrow
CAR	chimeric antigen receptor
CLL	chronic lymphocytic leukemia
CMV	cytomegalovirus
CR	complete revision
DC	dendritic cell
DFS	disease free survival
ECM	extracellular matrix
EGF-R	epidermal growth factor receptor
EV	extracellular vesicle
FC	flow cytometry
FDA	Food and Drug Administration
G-CSF	granulocyte-stimulating factor
GM-CSF	granulocyte-macrophage stimulating factor
GMP	good medical practice
Had	graft-versus-host disease
HLA	human leukocyte antigen
HSC	hematopoietic stem cells
HSCT	hematopoietic stem cell transplantation
ICPi	immune checkpoint inhibitor
IDO	indolamine 2, 3-deoxygenase
IFN	interferon
IL	interleukin
iPSC	induced pluripotent stem cell
KIR	killer cell immunoglobulin-like receptors
LAG	lymphocyte-activation gene
mAb	monoclonal antibody
MDSC	myeloid-derived suppressor cell
MHC	major histocompatibility complex
MIC	MHC class I-related
MMP	matrix metalloproteinase
NCR	natural killer receptor
NK	natural killer
OS	overall survival
PBMNC	peripheral blood mononuclear cell
PD1	programmed death
PD-L1	programmed death ligand 1
RNA-seq	RNA-sequencing
TAM	tumor-associated macrophages
TCR	T cell receptor
TGF-β	transforming growth factor β
TIGIT	T cell immunoglobulin and immunoreceptor tyrosine-based inhibitory motif
TMB	tumor mutational burden
TME	tumor microenvironment
TNF	tumor necrosis factor
TRAIL	TNF-related apoptosis inducing ligand
Treg	regulatory T cell
ULBP	UL-16 binding protein
VEGF	vascular endothelial growth factor
